# Supplementary material for: Complexome profiling of the Chlamydomonas psb28 mutant reveals TEF5 as an early PSII assembly factor
Source: Plant Cell. 2025 Mar 17;37(6):koaf055. doi: 10.1093/plcell/koaf055 (PMC12164590; doi:10.1093/plcell/koaf055)
Supplement: koaf055_Supplementary_Data [file koaf055_supplementary_data.zip › psb28_interactive.zip!/plots/intensity/qualityPlots/peptides/_Cre02.g093650.t1.2.html]

### Cre02.g093650.t1.2

Trivial name:   
SeqCoverage [median]: 42.80  
Mass: 15.01 kDa  
  
 

| Sequence | MissedCleavage | Mass [Da] | wtIntensity | muIntensity | Score |
| --- | --- | --- | --- | --- | --- |
| ISDNGDIEVNV | 0 | 1173.55 | 416.86 | 0.00 | 88.95 |
| KPLPVFQVR | 1 | 1082.66 | 0.00 | 5930.81 | 65.25 |
| LPNLPLVGK | 0 | 949.60 | 316.74 | 13095.64 | 89.30 |
| QVVEVGGQK | 0 | 942.51 | 8243.96 | 3492.86 | 118.32 |
| SAGGKQVVEVGGQK | 1 | 1342.72 | 2635.74 | 1351.28 | 138.02 |
| TALFTAEVK | 0 | 978.54 | 762.66 | 9571.53 | 81.30 |
| TALFTAEVKDK | 1 | 1221.66 | 0.00 | 316.58 | 58.68 |
| VSSTSALK | 0 | 791.44 | 17788.58 | 25049.45 | 108.92 |
|  |  |  | 30164.5 | 58808.2 |  |
